# A randomised control trial protocol of MuST for vascular access cannulation in hemodialysis patients (MuST Study): contributions for a safe nursing intervention

**DOI:** 10.1186/s12882-022-02842-3

**Published:** 2022-06-21

**Authors:** Ricardo Peralta, Anna Wammi, Manuela Stauss-Gabo, Óscar Dias, Helena Carvalho, António Cristóvão

**Affiliations:** 1 Lisbon School of Nursing, University of Lisbon, Lisbon, Portugal; 2 NephroCare Portugal, Fresenius Medical Care Portugal, Lisbon, Portugal; 3 Fresenius Medical Care, Global Medical Office, Bad Homburg, Germany; 4 School of Sociology and Public Policy, University Institute of Lisbon, Center for Research and Studies in Sociology, Lisbon, Portugal

**Keywords:** Arteriovenous fistula, Vascular access, Cannulation, Rope-ladder, Pain, Hemodialysis, MuST, Protocol, Randomised control trial

## Abstract

**Background:**

The vascular access preservation and the maintenance of a complication-free fistula remains an Achilles’ heel of hemodialysis in chronic kidney patients due to its substantial contribution to the morbidity and mortality. Systematic studies in the area of examining cannulation practices, achieving complication-free cannulation, and strategies to improve fistula survival are needed. For this reason, we consider it essential to create and investigate new methodologies for approaching fistula in patients on regular HD.

The Multiple Single Cannulation Technique (MuST) is based on the association between the rope-ladder (RL) using the arteriovenous vessel through progressive rotation, and the buttonhole (BH) since there are three specific cannulation sites for each cannulation day during the week. The MuST is simple to implement and seems to be a very promising technique in terms of patient safety. Previous studies already showed an arteriovenous fistula survival similar to RL but significantly higher than BH.

**Methods:**

This MuST study is a multicenter, prospective, non-blind, parallel-group, randomized controlled trial with the intervention group submitted to MuST and a control group undergoing the rope-ladder, up to 100 subjects for each group. Patients will be randomized 1:1 to one of two cannulation technique (CT), and the follow-up period of this study will be 12 months. Primary outcome is to evaluate the arteriovenous fistula survival rate at 12 months determined by the percentage of fistulas in use from the beginning of the study to the date of the first clinical intervention by angioplasty or vascular surgery, to maintain or restore patency (unassisted patency). Secondary outcome is to evaluate arteriovenous fistula survival rate at 12 month determined by the percentage of fistulas in use from the study start to the date of access abandonment due to dysfunction, patient abandonment, or death, treatment change modality or study end. We will also evaluate the assisted primary patency and include the following secondary outcomes associated with the cannulation technique: Infection, Hematoma, Aneurysm development, and pain.

**Discussion:**

The study will investigate whether fistula survival can be improved when using cannulation by MuST compared to the RL. MuST study will provide important information on fistula survival when cannulated by MuST but also information related to its use in fistulas previously cannulated by other CTs.

**Trial registration:**

ClinicalTrials.gov identifier NCT05081648 registered on 18 October 2021.

## Background

Despite technological advances in the treatment of chronic kidney disease (CKD), vascular access (VA) remains one of the main causes of comorbidities and hospitalization of hemodialysis (HD) patients [1, 2]. A functioning VA guarantees the efficiency of the treatment and represents the lifeline for these patients. In a systematic review, Casey et al. [3] concluded that patients on HD have an increased vulnerability, not only due to their underlying chronic disease, but also as they must maintain a functioning VA. Bodily intrusion fear of cannulation, disfigurement, threat of complications, potential VA failure and difficulty sleeping are some of the concerns expressed by patients. For some patients, the fear of cannulation is an impediment to the construction of a VA or leads treatment refusal [3].

Another serious complication potentially related to cannulation techniques is local infection of the VA or bacteremia. This complication has been found to be associated with the buttonhole cannulation (BH) technique [4, 5]. However, a meta-analysis performed by Chong Ren et al. [6] failed to demonstrate this increase in prevalence, due to heterogeneity of results.

Two systematic literature reviews and a meta-analysis [6–8] did not find conclusive results when comparing the rope-ladder (RL) and BH with relation to the bleeding time reduction, hematoma, hospitalization, interventions, or VA survival. According to the authors, these limitations are associated with the studies` poor quality and heterogeneity.

The VA is often described as a lifeline [9] that enables chronic kidney patients undergo HD as a replacement therapy for renal function, enabling their survival and maintainence of an acceptable quality of life. The health, safety, and well-being of the patient must be ensured when providing care. In this way, risks must be identified, monitored and, whenever possible, prevented. Successful cannulation of a VA depends on the choice of cannulation technique (CT), environmental influences, the skill of the nursing staff, practical experience, but also on the talent, confidence, and commitment of the caregiver. The arteriovenous fistula (AVF) CT is an invasive procedure and is not free from complications but should be performed in a way that does not cause further damage or injury beyond that resulting from the essential act of clinical intervention.

However, VA preservation and the maintenance of a complication-free AVF remains an Achilles’ heel [9, 10] due to its substantial contribution to the morbidity and mortality in this patient population. Thus, demanding studies in the area of examining cannulation practices, achieving complication-free cannulation, and strategies to improve AVF survival are needed [11].

MuST is a novel Multiple Single Cannulation Technique (MuST) [12] that is based on the association between the RL by using the arteriovenous vessel through progressive rotation and the BH. This cannulation technique uses three specific cannulation sites for each day during week, allowing the cannulation sites to heal during cannulation.

In a previous randomized controlled trial (RCT) published by Peralta et.al., three cannulation techniques (CT) MuST, RL and BH have been compared by using fistula not previously used by other CT [13]. The trial was conducted in 18 clinics that recruited 172 patients randomly assigned to three different cannulation techniques. The primary endpoint was AVF primary patency at 1 year. The results showed that MuST is simple to implement and seems to be a very promising technique in terms of patient safety [13, 14]. Moreover, a significantly higher AVF survival rate compared to the BH technique could also be shown in this trial.

Therefore, the aim of the MuST study is to investigate whether new methodologies of cannulation improve fistula survival in patients on regular HD.

With the MuST Study, we are going to use fistulas previously used by traditional CT. In addition to evaluating AVF survival, we intend to evaluate other secondary outcomes not evaluated in the previous study.

## Methods

This study is a multicenter, prospective, non-blind, parallel-group, randomized controlled trial with the intervention group undergoing MuST procedure and a control group undergoing the RL technique. Patients will be randomized 1:1 to one of two CT, MUST or RL, and the follow-up period of this study will be 12 months. For each treatment group up to 100 patients are planned to be included.

## Study Objectives

The aims of the MuST Study are to:

- Determine the AVF survival of patients submitted to MuST compared to those submitted to RL.
- Determine the AVF complication rate of patients submitted to MuST compared to those submitted to RL.
- Analyze the intensity of pain perceived by the patient with each cannulation technique under study.

### Study hypotheses

This study examines the following hypotheses:

- Does MuST allow 10% rate of greater AVF survival in patients on a regular HD program in private HD clinics than RL?
- Does MuST have a lower AVF complication rate in patients on a regular HD program in private HD clinics than RL?
- Do patients submitted to MuST perceive less pain than patients submitted to RL?

### Study population

#### Recruitment of the study population

Patients with end-stage kidney disease (ESKD) on a regular HD program will be recruited in 3 private dialysis clinics operating in Portugal. Decision on a study participation is made by the patient. The treading physician will inform the patient about the study and provides written information. If the patient is not willing to participate in the study by giving its written informed consent, the patient cannot be included in the study.

The patients who signed the informed consent and meet the inclusion and exclusion criteria, will be randomly organized into two groups – intervention and control groups. Each group will be stratified according to the following criteria: diabetes and AVF vintage to ensure that these variables is properly represented in the sample and avoid bias*.*

#### Inclusion criteria

Patients with AVF will be selected when:

- Are on a regular HD program with three weekly sessions;
- AVF has been in use for at least 8 weeks without incident;
- AVF with blood flow (Qa) ≥ 500 mL/min evaluated by thermodilution;
- AVF paths allow cannulations along the entire length of the vein with at least 6 cm of distance between bevels, or two distinct areas of 3 cm in length;
- Adult patients

### Exclusion Criteria

Patients with the following characteristics will be excluded:

- Those who decline to take part;
- Those who have undergone angiography or surgical intervention in the last 4 months in the AVF in use;
- Those who have undergone three or more interventions in the AVF in use;
- Those with use of anesthetic creams at cannulation sites.

### Intervention

For the study implementation, the key VA person and the principal investigator in each clinic will be involved and the following procedures will be carried out:

- Identification of all patients with AVF;
- Selection of eligible patients;
- Informing potential participants about the study and requesting informed consent;
- Participants’ randomization in a 1:1 ratio, according to MuST versus RL. Randomization will be performed centrally by the project manager and electronically using random sequence generator, two branches (columns) from RANDOM.ORG.

The key VA person and the nurse’s team will be carried out the following steps:

- Collect AVF photo, before the start of the study (baseline), at 6 months and at 1 year during the study;
- MuST cannulation technique – select and identify cannulation sites with a dermographic pen until their location is easily visible due to skin depigmentation; Two areas of arterial and venous cannulation will be created, with three cannulation points each at least 1 cm apart;
- Rope-ladder cannulation technique – create a diagram of the cannulation sites’ orientation. The diagram will be attached to each patient’s dialysis file for quick reference;
- AVF physical examination before each HD treatment with recording of the findings;
- The daily results of each patient’s assessment will be recorded using the computer tool VASACC (vascular access) and on an Excel spreadsheet;
- The nurse who performs the cannulation will assess the intensity of pain perceived by the patient;
- Pain will be assessed immediately after cannulation of the arterial and venous area according to the 10 cm long visual analog scale (VAS) which has the classification “No Pain” on the far left and “Extreme Pain” on the right [4, 17, 21];
- Whenever there is a referral to the Vascular Access Center (VAC) for angiography or vascular surgery, a record will be made in the patient’s clinical file in the EUCLID database;
- After the intervention in the VAC, a record of the intervention per patient will be made in the Vascular Access OnLine database (AV OnLine). This registration will be performed by the nephrologist or vascular surgeon, depending on which intervention is performed. This information will then be transcribed into the Excel data collection Spreadsheet;
- Patients will be followed until access thrombosis, abandonment due to AVF dysfunction or surgical intervention with anastomosis alteration, patient abandonment from the study, transfer to another clinic, hospitalization, death, change of treatment modality or study end;
- For the variable pain intensity, time to hemostasis, peri-needle bleeding, Qa, dialysis dose (*sp*kt/V) and substitution volume will be evaluated at 3 time periods only: before study start, at 6 months, and at 12 months;
- Presence of scab at the cannulation site and the ease of identifying the cannulation site will be evaluated at the same time periods, but only for CT MuST;
- Monthly meetings will be held with the head nurse and key VA person, and additional meetings whenever necessary. All situations deviating from normality there will be discussed. Verification and collection of data.

In this clinical trial, we use a new fistula approach, because of which it is necessary to carry out training and raise awareness of nurses to assess and record the variables under study. Thus, it is not possible to blind the participants nor the nurses who perform the intervention.

Physicians performing angioplasty or vascular surgery will not be aware of the selected patients.

We will involve more than forty nurses, more than two hundred patients and ultimately, this can lead to improved evidence on fistula preservation.

### Study Outcomes

#### Primary outcome

As a primary indicator, we consider AVF survival rate at 12 months and determined by the percentage of fistulas in use from the time-zero of study enrollment to the date of the first clinical intervention by angioplasty or vascular surgery, to maintain or restore patency – "unassisted patency" [15, 16].

To assess the variable "AVF survival" we consider the following criteria:

- Fistula that is successfully cannulated with two needles, arterial and venous, that allows a prescribed blood flow of at least 300 mL/min and that allows for an adequate treatment will be considered a functioning AVF [17];
- Fistula used without success will be considered a dysfunctional AVF, whether it has patency or not [15];
- It will be considered AVF abandonment on the day that access is considered permanently unusable or not suitable for cannulation [17];

Referral for endovascular intervention [15, 16] will be based on two of the following factors of AVF dysfunction:

- Decreased AVF blood flow (Qa) (assessed by thermodilution and <400 mL/min);
- Increased hemostasis time (>10 min);
- Cannulation failure: failure or inability to insert dialysis needles [17];
- Decreased dialysis efficacy: low HD dose, *sp*Kt/V <1.2 [17] or substitution volume <21L. A parameter to quantify the HD adequacy (*sp*Kt/V) will be calculated based on ionic dialysance obtained by the integrated module Online Clearance Monitor (OCM®) from the 5008 CorDiax machine, and “*V*” was derived from total body composition assessment with a bioimpedance device (Body Composition Monitor - FMC) [18];

Or changes in the physical examination (changes in thrill, abnormal development of aneurysm, or progressive increase in edema in the AV limb) and plus one of the factors described above.

Referral for surgical intervention [15] will be based on one or more indicators of AVF dysfunction:

- Rupture of the AVF wall;
- AVF thrombosis;
- Progressive development of aneurysm;
- Acute bleeding: AVF bleeding requiring surgical intervention;
- Local infection of the AVF;
- Deficient distal perfusion with signs of ischemia.

### Secondary outcome

We will evaluate the assisted primary patency. As an outcome, we considered AVF survival rate at 12 month and determined by the percentage of fistulas in use from the time-zero the study enrolment, to the date of access abandonment due to dysfunction, patient abandonment, or death, treatment change modality or study end [15, 17, 19]. For the study, we will consider the frequency of interventions, both endovascular and surgical, to maintain functioning access [17].

We will also include the following secondary outcomes associated with cannulation:

- Inflammation signs at the AVF cannulation site, defined by the presence of one or more signs: redness, edema or local exudate [4];
- AVF local infection, defined by the presence of exudate at the cannulation site with a positive bacteriological culture;
- Bacteremia related with AVF and confirmed with a positive blood culture (describe if swab was performed and/or infection treated with antibiotics);
- Hematoma or infiltration: an incident that occurs during cannulation that can result in local infiltration, edema, or pain, which can be treated with local ice, but re-cannulation is possible [16];
- Time to hemostasis: time to stop bleeding after needle removal, with up to 10 min considered normal [4];
- Peri-needle bleeding: bleeding from the puncture site during treatment and requiring nursing intervention;
- Aneurysm development: segment dilatation of the arterialized vein at three times the diameter of the segment considered normal, which means a segment with a width of ≥ 18 mm [20]. An increase in the existing aneurysm will be considered when the vein presents an increase in its diameter of ≥ 5 mm.
- Local pain related to CT;
- Presence of scab at the cannulation site;
- Easy to identify cannulation site.

### Outcome parameters

For the sample characterization, we will describe the sociodemographic characteristics, comorbidities and laboratory data immediately before the start of the study (baseline). We will also describe the interventions already carried out in active AVF within the last year (T0).

### Data collection instrument

- To collect the data, an Excel Spreadsheet was created, consisting of 5 sheets:

Patient baseline; Follow-up data changes; Follow-up data semester; AVF intervention and Outcome.

- The data resulting from the evaluations will be collected from the VASACC and EuCliD databases and recorded on the Excel data collection Spreadsheet.
- A computer file will be created to store the patient’s VA photos. Each photo will be given an identification number.

### Data usage

- The use of the information collected is exclusively for academic purposes and the results of the study may be published in peer-reviewed scientific journals and presented at relevant national and international meetings, preserving the identity of all participants

Pain assessment scale:

- Visual analog scale (VAS).

Pain will be assessed immediately after cannulation of the arterial and venous area according to the 10 cm long visual analog scale (VAS) which has the classification “No Pain” on the far left and “Extreme Pain” on the right [4, 17, 21].

The values obtained will later be registered on the Excel data collection Spreadsheet.

### Statistical analysis

Data will be analyzed according to the “intention-to-treat” [15]. The continuous variables of the “baseline” will be expressed through measures of location, dispersion and the categorial variables will be summarized using absolute and relative frequencies. Descriptive comparisons and tests will be performed between groups at baseline, at 6 and 12 months. To compare two groups by continuous variables, parametric tests will be used, namely the t-test, or nonparametric tests if normality is not assumed.

To analyse relationship between categorical variables, the Chi-square test (χ^2^) will be conducted, or Fisher’s exact test when appropriate.

The primary factors to assess fistula survival will be calculated over time from baseline to time requiring intervention (no patency assistance) or withdrawal from the study. In this case we will use the Kaplan–Meier survival curves.

For secondary factors, such as the frequency of hematoma, signs of infection, local infection or bacteremia, and thrombosis, the number of events per 1000 days of AVF will be calculated [16].

Results will be considered significant when *p* < 0.05. All the statistical analysis will be performed using SPSS (version 23; IBM, Armonk, NY, USA).

## Discussion

Any insertion of a needle is an invasive procedure that induces tissue damage, pain, and when performed incorrectly or improperly, has serious consequences, both immediate and subsequent, on patient safety. Studies report that area CT should be avoided as it creates aneurysmal development in the cannulation sites and subsequent VA failure. Regarding the other two CT, RL versus BH, results are controversial in relation to pain perception and assessment tools and methodologies are heterogeneous. On the one hand, buttonhole CT shows a reduction in the development of aneurysms, on the other hand, it is associated with an increased risk of infection at the cannulation site, and bacteremia. Therefore, we believe that it is necessary to create new approaches to AVF in patients on a regular HD program. Previous RCT [13] using MuST showed an AVF survival was similar to RL, however it was not evaluated assisted primary patency and other secondary outcomes such as pain that are included in this study. MuST study will provide important information on fistula survival cannulated by MuST and as well as other information related to its ease of use in fistulas previously cannulated by other CTs.

## Data Availability

Only the investigators will have access to the data. The datasets generated and analyzed during the current study are not publicly available but are available from the corresponding author on reasonable request. A dissemination plan has been developed which is directed towards different stakeholders: a) Patients with chronic diseases, b) Health professionals, c) Politicians, d) Patient organisations, e) Scientific circles.

## Abbreviations

AVF: Arteriovenous fistula
BH: Buttonhole
CT: Cannulation technique
CKD: Chronic kidney disease
ESKD: End-stage kidney disease
EuCliD: European Clinical Data Base
FMC: Fesenius Medical Care
HD: Hemodialysis
LMWH: Low-molecular-weight heparin
MuST: Multiple single cannulation technique
OCM: Online Clearance Monitor
Qa: AVF blood flow
RCT: Randomized controlled trial
RL: Rope-ladder
spkt/V: Single-pool urea kinetic modelling
UFH: Unfractioned heparin
VA: Vascular access
VAS: Visual analog scale
VASACC: Vascular access database
